# Epidemiology of rubella virus cases in the pre-vaccination era of Ethiopia, 2009–2015

**DOI:** 10.1186/s12889-016-3841-z

**Published:** 2016-11-18

**Authors:** Mekonen Getahun, Berhane Beyene, Kathleen Gallagher, Ayesheshem Ademe, Birke Teshome, Mesfin Tefera, Anjelo Asha, Aklog Afework, Esete Assefa, Yoseph HaileMariam, Yonas HaileGiorgis, Hiwot Ketema, Dejenie Shiferaw, Ayenachew Bekele, Daddi Jima, Amha Kebede

**Affiliations:** 1 Ethiopian Public Health Institute, Arbegnoch Street, P. O. Box 1242, Addis Ababa, Ethiopia; 2 WHO Country Office, Addis Ababa, Ethiopia

**Keywords:** Ethiopia, Rubella, Pre-vaccine era, 2009–2015

## Abstract

**Background:**

Rubella is a common mild rash illness caused by rubella virus. The majority of infections occur in children and young adults. The infection is the cause of a serious birth defect known as Congenital Rubella Syndrome (CRS) when a woman acquires infection early in pregnancy. Ethiopia has not yet established rubella virus surveillance and has not yet introduced rubella vaccine into the routine immunization program. We characterize the epidemiology of laboratory confirmed rubella virus cases collected through measles surveillance from 2009 to 2015 to better understand the burden of the disease in the country.

**Methods:**

A descriptive analysis was made to characterize rubella cases reported through the national measles case based surveillance system. The measles case definition was used to capture potential rubella cases. A suspected measles case was a person with generalized rash and fever with cough, or coryza or conjunctivitis. Those cases whose sera were negative for measles IgM antibodies were tested for rubella IgM antibody. A confirmed rubella case was a person who tested positive for rubella IgM. Only laboratory confirmed rubella cases were analyzed in this article.

**Results:**

Between 2009 and 2015, a total of 28,284 serum/plasma samples were collected and tested for measles IgM antibody and 11,151 (39.4%) were found positive. A total of 17,066 measles IgM negative or indeterminate samples were tested for rubella virus IgM and 2615 (15.3%) were found positive during the same period. Of 2615 confirmed rubella cases, 52.2% were females. The age of confirmed cases ranged from one month to 42 years with a mean age of 7.3 years. Three-fourth of all confirmed rubella cases were aged less than 10 years. The number of laboratory confirmed rubella cases linearly increased from 83 in 2009 to 856 in 2013 but dropped to 222 and 319 in 2014 and 2015 respectively. Higher number of cases occurred in the hot dry season (January through June) and in the central and western part of Ethiopia with 127 lab-confirmed outbreaks in the study period.

**Conclusions:**

Based on our analysis, rubella was found to be endemic throughout Ethiopia. Children below the age of 10 years were the most affected. The burden of rubella cases varied from year to year but had a seasonal peak in March. To better understand the magnitude of rubella prior to vaccine introduction, establishing rubella surveillance system, conducting sero-prevalence studies among child bearing age females and establishing CRS sentinel surveillance among young infants are critical.

## Background

Rubella, also known as German measles, is a mild self-limited viral illness which shares similar symptoms of rash and fever with measles infection. Rubella usually occurs during childhood, but infection occurring just before conception and during early pregnancy may result in miscarriage, fetal death, or congenital defects of newborns known as Congenital Rubella Syndrome (CRS). The defects associated with CRS affect all organs of the body; complications of the thrombocytopenia being fatal. Affected children may shed the virus for a year or more in pharyngeal secretions and urine. The effects of rubella virus on the fetus are largely dependent on when in pregnancy the infection occurs; the younger the fetus when infected, the more severe the illness as the fetus is early in organo-genesis. The highest risk of CRS is found in countries with high rates of susceptibility to rubella among women of child bearing age. Rubella occurs worldwide in non-vaccinated population with varying incidences of outbreaks [1–4].

Rubella is caused by rubellavirus in the genus rubivirus of togaviridae family, which replicates in the mucus (nose and throat) of infected persons and spreads by direct contact with susceptible hosts through droplet sprays during coughing and sneezing. The infection is vaccine preventable. Both vaccine and natural infection result in life long immunity [4].

When a pregnant mother is infected with rubella virus during the first trimester of pregnancy, the risk of developing CRS in the fetus is up to 90% [4, 5]. Prevention of CRS is the main reason for rubella vaccination programs [6]. Although rubella is vaccine preventable and an effective single dose vaccine is available, many developing countries, including Ethiopia. have not yet introduced it in their routine immunization system.

In African countries, including Ethiopia, information on rubella epidemiology is very limited [5]. In Ethiopia, rubella surveillance has not yet been established [7]. The measles case-based surveillance system, established in 2004, has provided an opportunity for detection of rubella specific immuno-globulin M (IgM) antibody among persons with rash illness who are not positive for measles IgM.

This article summarizes the results of rubella testing through the measles case-based surveillance system and provides an epidemiological description of the laboratory-confirmed rubella cases in Ethiopia during 2009–2015 (pre-vaccine era) as an important step in generating baseline data on rubella infection and in the development of a rubella control program in Ethiopia.

## Methods

### Study setting

Ethiopia is the second most populous country in Africa with a population of 98 million. Children under 5 years of age make up 12.5% of the population. 90% of the population has access to formal health care services. Life expectancy at birth was 55 years, and the total fertility rate was 5.3 children per woman of child bearing age. The country has a surface area of 1.1 million square kilometres and is administratively divided into 9 regional states and 2 city administrations. There are more than 80 linguistic groups in Ethiopia. Djibouti, Eritrea, the Republic of Sudan, the Republic of the South Sudan, Kenya, and Somalia border the country [8, 9].

### Case definition

A suspected measles case was defined as any patient who presented with fever, generalized maculopapular rash, and either cough, or coryza, or conjunctivitis regardless of age and sex. Blood samples are collected on suspected cases and all sera are tested for the presence of measles IgM antibody. This surveillance system provided a platform for identifying suspected rubella cases. Suspect measles cases with sera negative for measles IgM antibody are further tested for rubella. Laboratory confirmed rubella cases were patients who had a positive rubella IgM test results by enzyme linked immunosorbent assay (ELISA) technique. A confirmed rubella outbreak was defined as a cluster of 5 or more IgM confirmed rubella cases occurring within a month period within a district [10].

### Sample collection

Blood samples were collected from all suspected measles cases during 01 January 2009–31 December 2015. Demographic and clinical information about the patient was captured through the case based reporting form (CRF). For measles and rubella testing, samples were transported in a cold box to Ethiopian National Measles and Rubella Laboratory located at the Ethiopian Public Health Institute (EPHI), Addis Ababa, Ethiopia.

### Laboratory method

First, all samples were tested for measles specific IgM antibody and those samples having negative or two sets of indeterminate (equivocal) measles results were tested for rubella specific IgM by indirect ELISA technique using a commercially available standard kit (Siemens Diagnostics, Marburg, Germany).

A serum/plasma sample of 5 μl volume was diluted in a 1:21 ratio using diluting plate (two wells for one sample). 150 μl of diluted sample was then transferred to a rubella antigen coated test plate and incubated at 37 °C for an hour. Then the plate was washed with an ELISA plate washer to remove unattached antibodies and debris, and 100 μl enzyme labeled anti-human IgM working solution was added to the wells and incubated at 37 °C for an hour. After washing, a substrate-chromogen working solution was added and incubated at room temperature for 30 min to allow the labeled enzyme (if any) break the substrate and give color through the chromogen. Finally, a stop solution was added to stop the substrate-enzyme reaction and the optical density (OD) of the wells were read with an ELISA reader.

Based on the protocol, the read out was recorded in two programs of the machine. One, the OD value of each well was given (antigen and control OD). Second, the calculated change in OD of each sample (antigen well OD minus control well OD) was recorded. Those samples having a change in OD value of >0.2 were registered as positive and those <0.1 were negative for rubella virus IgM. Samples with a change in OD between 0.1 and 0.2 were recorded as indeterminate (equivocal). All samples were tested once for rubella IgM.

### Data analysis

The laboratory results and patient information from the case report form were entered into an Epi-info based electronic database. The case-based surveillance data were regularly consolidated, cleaned, analysed and disseminated to stakeholders for action including the Federal Ministry of Health (FMOH), World Health Organization (WHO) Ethiopia Country office and WHO Regional Office for Africa. Data for the purpose of this study were extracted and analysed by Epi-Info software version 3.5.4.

### Quality assurance

The Ethiopian National Measles and Rubella laboratory is member of the global WHO vaccine-preventable diseases laboratory network, As such, it is subjected to periodic quality control checks and accredited annually in order to deliver credible results for the program. All the equipment and materials of the laboratory were supplied by WHO. Standard operating procedures (SOPs) and job aids are available for lab activities. The lab receives external quality assessment (EQA) samples once a year and sends 10% quality control (QC) samples quarterly. To check the validity of each run, kit and in house control materials (negative and positive) were used and patient results were reported only for a valid run. The lab performance of ≥95% accuracy for both EQA and QC in the study period is a witness for credible result delivery.

## Results

During the 7 year study period, a total of 28,284 samples were received and tested for measles IgM antibody, 2009–2015, Ethiopia. Of these samples, 11,151 (39.4%) were found positive for measles IgM; 16,314 and 819 were found to be negative and indeterminate respectively. Of the total 17,066 negative and indeterminate samples tested for rubella virus specific IgM, 2,615 (15.3%) were positive with 12,904 and 1547 negative and indeterminate, respectively.

Most (97.3%) of the samples were collected within 14 days of rash onset and 93.3% reached the national laboratory within three days of collection. Results were available within seven days of receipt for 71% of the samples tested.

The number of specimens tested for rubella varied from year to year with the lowest (1974) in 2009 and the highest, (3581) in 2013. The number of rubella cases identified and the rubella positivity rate increased from 83 (4.2%) in 2009 to 856 (23.9%) in 2013 (Table 1).

**Table 1:** Characteristics of Lab-Confirmed Rubella Cases, Ethiopia, 2009–2015

Variables	Category	Rubella IgM Test Result			Total samples tested for Rubella IgM
		Positive	Negative	Indeterminate	
Sex	Male	1246	6940	717	8903
	Female	1364	5900	826	8090
	Unavailable	5	64	4	73
Year	2009	83	1802	89	1974
	2010	131	1976	170	2277
	2011	198	1354	242	1794
	2012	806	2037	431	3274
	2013	856	2402	323	3581
	2014	222	1714	149	2085
	2015	319	1619	143	2081
Age Groups	<1 Year	63	1038	38	1139
	1–4 Years	793	4518	435	5746
	5–9 Years	1103	3657	560	5320
	10–14 Years	413	1862	277	2552
	≥15 Years	203	1652	209	2064
	Age Not Available	40	177	28	245
Regional State and City Administrations	Addis Ababa	537	1505	299	2341
	Afar	35	163	25	223
	Amhara	500	2312	287	3099
	Benshangul-Gumuz	72	223	28	323
	Dire-Dawa	22	69	8	99
	Gambella	25	34	9	68
	Harare	15	110	8	133
	Oromia	765	5873	582	7220
	Somali	6	170	7	183
	SNNPR	531	1962	235	2728
	Tigray	107	483	59	649
Total		2615	12904	1547	17,066

**Fig. 1:**
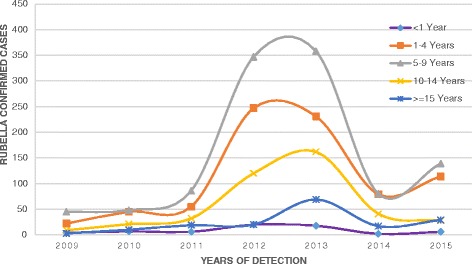
Trend of laboratory confirmed rubella cases by year and age group, 2009-2015, Ethiopia

**Fig. 2:**
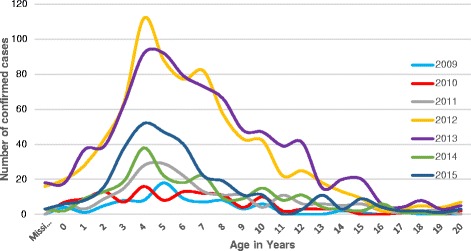
Age specific incidence of Rubella confirmed cases, 2009–2015, Ethiopia

The highest (20.7%) rubella IgM positivity rate and the highest number of positive cases (1,103) were among children aged 5–9 years (Table 1). The number of rubella cases increased among all age groups from 2009 to2013 and decreased in 2014 and 2015 (Fig. 1). Of the confirmed rubella cases, age was not specified for 40 cases. The number of laboratory confirmed rubella cases rises at 3–6 years of age and then declines with increasing age (Fig. 2).

The majority of the lab-confirmed rubella cases were detected from the central and western part of the country and was highest from the Oromia Region (29.3% of all confirmed cases) followed by Addis Ababa (20.5%), SNNPR (20.3%) and Amhara Region (19.1%) (Table 1). 89% of all confirmed rubella cases were from these four regions with the highest proportion of positives (22.9%) from Addis Ababa and the lowest (10.6%) from Oromia. These regions are the four most populous regions in the country.

**Fig. 3:**
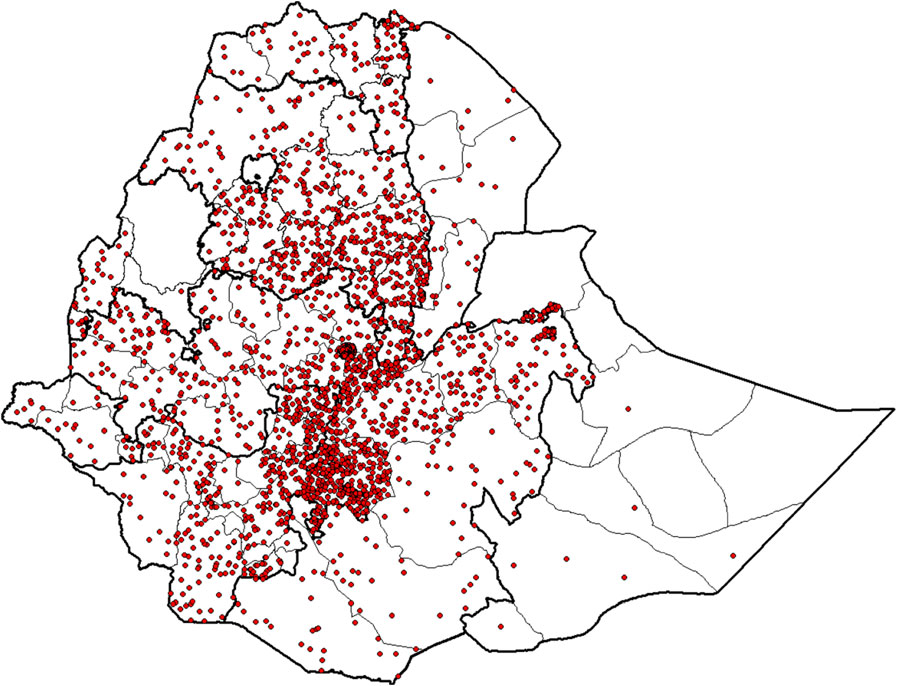
Spot map of Ethiopia showing geographical distribution of laboratory Confirmed Rubella cases, 2009–2015

The number of confirmed rubella cases was higher from the highly populated central region (near to national laboratory) and Western part of the country and lower from the Eastern part of the country where nomadic communities live sparsely (Fig. 3). Sixty-three point five percent of confirmed cases occurred in 2012 and 2013 (Table 1).

In general, a minimum of 127 rubella virus outbreaks were identified by laboratory confirmation in Ethiopia during 2009–2015. The number of outbreaks increased dramatically from 3 in 2009 to 38 in 2013 and dropped to 23 in 2015. The outbreaks were 3, 7, 8, 28, 38, 20 and 23 by count in 2009, 2010, 2011, 2012, 2013, 2014 and 2015 respectively.

**Fig. 4:**
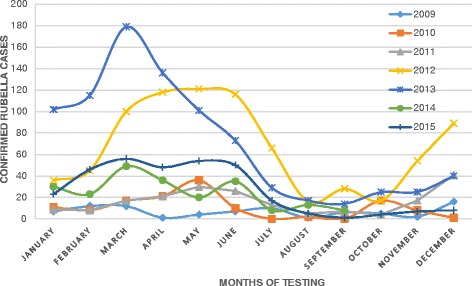
Seasonal pattern of rubella infection, 2009–2015, Ethiopia

There was a seasonal pattern in the occurrence of laboratory confirmed rubella cases, with peaks in the hot dry months (January to June) of Ethiopia. There was a sharp decline in August and September of each year as shown in Fig. 4.

## Discussion

Currently, rubella vaccination is not part of child routine immunization services in Ethiopia and a standalone surveillance system for rubella and CRS does not exist. In the major urban centers, some private practitioners provide rubella containing vaccine (RCV) to infants at 9 months of age or older in the form of measles-rubella vaccine, but the coverage is unknown among the general population as the services is not monitored yet. Little attention has been given to rubella as it is not considered a killer disease. The major impetus behind rubella vaccination and rubella related studies is to reduce the risk of CRS. Unfortunately, there is no recent data on the incidence of CRS in Ethiopia to guide evidence-based decision making for rubella vaccine introduction.

During 2009–2015, 2615 laboratory-confirmed rubella cases were identified from all 9 regions and 2 city administrations of Ethiopia. This is almost three times the 992 confirmed cases of rubella for a previous 6 year period in Ethiopia reported by Kassahun et al. [11]. This might be explained by improvement in measles surveillance over time or to a real increase in rubella cases since there is no any intervention activity. Of the confirmed rubella cases, 91% were reported among children <15 years of age, indicating that rubella is mainly an illness of childhood.

Of 17,066 laboratory tested cases, 15.3% were confirmed rubella positive cases. The proportion of rubella positive cases increased from 4.2% (83) in 2009 to 23.9% (856) in 2013 but dropped to 10.6% (222 cases) and 15.3% (319 cases) in 2014 and 2015 respectively. This was higher than previous findings from Ethiopia [11] and Abia State of Nigeria [12] and was lower than a finding of 37.6% from Zimbabwe [13]. The increasing number of laboratory confirmations might be related with improvement in surveillance sensitivity, quality and coverage.

Our finding in this study revealed that children aged 5–9 years were the most affected by rubella in Ethiopia. This was in agreement with previous findings from Ethiopia [11], Nigeria [12], and Kenya [14]. In an outbreak report from Poland, young male adults were the most affected [15]. In our analyses both sexes were equally likely to have rubella. About 93.1% of rubella confirmed cases were among <15 years old children and rubella reached its peak incidence in children between 3 and 6 years of age. This was similar to finding from a study in Zimbabwe [13]. The low incidence of rubella cases in persons older than 15 years is likely due to natural infection and development of immunity against rubella at earlier ages.

In this article, the highest rubella positivity rate was from Addis Ababa, the capital of Ethiopia. Similar high positivity rate of cases was found in Harare, the capital of Zimbabwe [13]. This may be related to the higher population density in cities that favors increased transmission. In addition, the number of lab-confirmed rubella virus cases was also higher in the central and western parts of the country. This may be related with high population density and short distance from the national laboratory that fosters sample transportation, Fig. 3. The lower incidence of rubella in the eastern part of the country (Somali and Afar Regions) might be related with the low population density in these areas which reduces the probability of disease transmission [8].

In our finding, rubella infection showed a clear seasonal pattern, higher in the hot dry season of Ethiopia (February to June) reaching a peak in March with the lowest number of cases occurring from July through September. This seasonal pattern was similar to findings from previous studies in Ethiopia [11] and Kenya [14]. Most outbreaks occurred in the hot dry season of the country.

The proportion of samples with equivocal test results in this finding was higher. This is true as all samples were tested once for rubella virus IgM antibody and those producing equivocal test results were not retested due to a lack of resources as retesting is advised for countries at rubella elimination phase [10].

On average, there were 18 annual outbreaks of rubella in Ethiopia and more than 63% of confirmed cases and 52% of confirmed outbreaks occurred in 2012 and 2013. This was demonstrated by a large rubella virus outbreak reported from Benshangul-Gumuz Regional state of Ethiopia that affected 7,269 people in 2012 and 2013 [16]. This outbreak situation is expected to continue until the country introduces rubella vaccine into the routine childhood immunization schedule.

The findings of this study are subjected to several limitations. First, the case definition to detect rubella cases was designed for measles cases and as a result may under-estimate the true burden of rubella in the country. Second, since up to 50% of rubella cases are asymptomatic, the case definition used would not be able to identify all rubella cases. Cases without symptoms, mild symptoms or without a rash would not have been identified. Thirdly, our analysis was not able to determine the prevalence of current rubella infection nor the immune status of reproductive age females in order to predict the risk of CRS in the population. Finally, we were unable to identify any epidemiologically-linked or clinically confirmed rubella cases during the study period as there is no ongoing rubella- specific surveillance. This would result in a decrease in the number of reported rubella cases and outbreaks in the country.

## Conclusion

We found that rubella was endemic throughout Ethiopia and children below the age of 10 years were the most affected. The number and proportion of rubella positive cases increased from 2009 to 2013 and there was a clear seasonal pattern of rubella infection which reached its peak in March.

To better understand the magnitude of rubella and to inform decisions about rubella vaccine introduction, establishing rubella surveillance system, conducting sero-prevalence studies among child bearing age females and establishing CRS sentinel surveillance among young infants are critical.

## Abbreviations

°C: Degree Celsius
CRF: Case-based reporting form
CRS: Congenital rubella syndrome
ELISA: Enzyme linked immuno-sorbent assay
EPHI: Ethiopian Public Health Institute
EQA: External quality assessment
FMOH: Federal Ministry of Health
IgM: Immuno-globulin M
OD: Optical density
QC: Quality control
RCV: Rubella Containing Vaccine
SERO: Scientific and Ethical Review Office
SNNPR: Southern Nation Nationalities and Peoples Region
SOP: Standard operating procedure
WHO: World Health Organization
μl: Micro-litre
